# Marginalised social groups differentials in nutritional status (BMI) among reproductive-aged women in West Bengal

**DOI:** 10.1186/s12889-023-15635-6

**Published:** 2023-05-10

**Authors:** Sourav Biswas, Koushik Roy Pramanik, C. J. Sonowal

**Affiliations:** 1 Department of Population & Development, International Institute for Population Sciences, Deonar, Mumbai, 400 088 India; 2 Department of Biostatistics & Epidemiology, International Institute for Population Sciences, Deonar, Mumbai, 400 088 India; 3 Professor, Centre for Study of Social Exclusion and Inclusive Policies, Tata Institute of Social Sciences, Deonar, Mumbai, 400 088 India

**Keywords:** Body mass index, NFHS-5, Cross-sectional study, Reproductive health, Nutrition, Scheduled population

## Abstract

**Background:**

SCs and STs, historically marginalized communities in India, have been subjected to social and economic discrimination for centuries. Despite affirmative action policies, these communities face systemic discrimination and exclusion in various aspects of their lives. Poor health conditions among SC and ST women are caused by insufficient consumption of nutritious food, leading to undernutrition and related health issues. To address gaps in the literature regarding the nutritional status of these women, this study aims to compare the BMI of SC and ST women in West Bengal and investigate the factors affecting their BMI. The study's findings can inform targeted interventions to improve the nutritional status of SC and ST women in West Bengal and reduce disparities in their health outcomes.

**Materials and methods:**

This study analyzed data from the National Family Health Survey (NFHS-5) to examine the distribution of underweight and  non-underweight SC and ST women in West Bengal. The sample included 5,961 non-pregnant reproductive-aged SC women and 1,496 non-pregnant reproductive-aged ST women. A binary logistic regression model was used to determine how background characteristics affect the nutritional status (BMI) of respondents, while a multivariate decomposition analysis was conducted to identify the covariates contributing to the nutritional status difference between SC and ST women. QGIS 2.18.25 software was utilized to map the spatial distribution of underweight and non-underweight SC and ST reproductive-aged women.

**Results:**

This study examines the nutritional status and differential background characteristics among SC and ST women in West Bengal, India. Results show that undernutrition affects the ST population more than the SC population, with age, marital status, religion, place of residence, educational status, and wealth quintile being significant factors affecting nutritional status. Food and dietary habits also impact nutritional status, with milk or curd, pulses or beans, dark green leafy vegetables, eggs, and fish being associated with lower rates of underweight. Binary logistic regression analysis reveals significant associations between socio-demographic factors and underweight status among SC and ST women. Socio-demographic factors were found to be the major contributors to the gap between SC and ST women, followed by food and dietary factors. The study highlights the need for targeted interventions to improve the nutritional status of marginalized communities like SC and ST women in West Bengal.

**Conclusion:**

The study highlights a significant population suffering from underweight in West Bengal, with socio-economic factors and dietary habits significantly contributing to the nutritional gap between SC and ST reproductive-aged women. Policy implications suggest targeted interventions to improve access to education and employment opportunities and promote a healthy and balanced diet to reduce the gap. Future studies could explore vulnerability risks of these domains.

## Background

### Definition

The Indian Constitution provides affirmative action policies to uplift historically marginalized communities in India [1]. These communities, known as SCs and STs, have been subjected to social and economic discrimination for centuries. The Indian Constitution identifies SCs and STs as specific groups that require special protection and support to overcome these longstanding inequities [2]. The Hindu caste system divides society into four castes: Brahmins (priests), Kshatriyas (warriors), Vaishyas (traders), and Shudras (menial workers). The castes of *Ati Shudras* (those performing the most impure or dirty menial tasks) were identified as being outside the fourfold caste structure, and these 'outcastes' are now known as SC since they have been listed in the specific scheduled of the Indian Constitution. ST are also separate from mainstream Hindu society, with distinct lifestyles, dialects, and cultural traditions different from India's established faiths and traditionally they reside mainly in the forest, hilly, and mountainous locations [3]. The Indian government has instituted various policies and programs to promote the socio-economic development of SCs and STs, including quotas in educational institutions and government jobs, reserved seats in elected bodies, and targeted welfare schemes. Despite these efforts, SCs and STs continue to face systemic discrimination and exclusion in various aspects of their lives, including education, employment, and healthcare [3–5].

### Population distribution

#### India

As per the latest census conducted in 2011, SCs constitute approximately 16.6% of India's population, while STs account for around 8.6% of the total population. These communities are spread throughout the country, with the highest concentration of SCs found in the states of Uttar Pradesh, West Bengal, and Bihar. The states with the highest ST populations are Madhya Pradesh, Maharashtra, and Odisha [6–8].

#### West Bengal

West Bengal is one of 28 states and eight union territories that make up India. West Bengal has significant socio-economic, demographic, and geographic differences. As per the Census of India (2011), the state has 23.51% share of the SC population and 5.8% of the ST population of the total population, ranking it as the second most populated SC state and ninth most populated ST state in India [8]. Despite constitutional safeguards and affirmative action policies, SCs and STs in West Bengal continue to face significant social, economic, and health disparities. In particular, the nutritional status of women belonging to these communities is often poor, with higher rates of undernutrition and related health issues. Understanding the health and nutritional status of SC and ST women in West Bengal is crucial for policymakers and researchers seeking to address these disparities and promote the health and well-being of all members of society.

### Nutritional status among reproductive women in the World, India, and West Bengal with a special focus on the SC and ST populations

Underweight among reproductive women is a significant global health issue, and India has a higher prevalence than the global average, with approximately 22% of women being underweight [9]. The situation is even more critical in West Bengal, where an estimated 33% of women are underweight [10]. Women from SC and ST communities are particularly vulnerable to this problem due to their higher likelihood of experiencing poverty, food insecurity, and poor health outcomes. Insufficient consumption of nutritious food is a significant factor contributing to poor health conditions among SC and ST women, leading to profound health implications such as undernutrition [11, 12]. Recent reports from the National Family Health Survey indicate that 13% of women in India are underweight, and one-third of SC and ST women fall into this category [10]. Although there has been a gradual decline in underweight trends, the proportions of underweight women in India, particularly from marginalized communities, remain problematic [13]. Recent studies suggest that 25.3% of ST women and 31.7% of SC women in India have faced malnutrition, indicating the urgent need for effective interventions to address this global health issue [7, 14].

### Relevance of the study and objective

SC and ST women in India have been reported to suffer from a lack of proper food intake, which can negatively affect their health and well-being [15, 16]. Understanding the nutritional status of these women is crucial for developing effective public health policies and programs that can address their specific needs. Body Mass Index (BMI) is a widely used measure of nutritional status that can provide valuable insights into the health of individuals and populations, including SC and ST women. However, there needs to be more research that examines the geographical differences and the impact of bio-demographic, behavioural, food, dietary pattern and household characteristics on the nutritional status of SC and ST women in West Bengal.

Several studies have been conducted on the health-related issues of SC and ST women, such as undernutrition, food and dietary patterns, and child and women's health [17–20]. However, there still needs to be a gap in the literature regarding comparing the nutritional status of SC and ST women, especially in West Bengal. Previous studies have also overlooked the methodological, conceptual, and theoretical gaps that can limit our understanding of the nutritional status of these women.

To address these gaps, this study aims to compare the BMI (underweight and non-underweight) of SC and ST women of West Bengal and investigate the factors that affect their BMI. The study has used decomposition analysis to identify the relative contributions of various factors to the observed differences in BMI among SC and ST women. The findings of this study can inform the development of targeted interventions that can improve the nutritional status of SC and ST women in West Bengal and reduce the disparities that exist in their health outcomes.

## Data and methods

### Data

Data for this study were gathered from fifth round of the National Family Health Survey (2019–2021). The International Institute for Population Sciences (IIPS), Mumbai, served as the nodal agency, conducted this nationwide sample survey that considered various socio-economic, demographic and health indicators at the district level. The NFHS-5 study is unique because it provides accurate data on multiple variables relevant to mother and child health, such as nutrition [21]. The study was done on women aged 15 to 49 years. In India, NFHS-5 contains a nationwide representative sample of 6,36,699 households, 7,24,115 women aged 15–49 years, and 1,01,839 males aged 15–54 years.

The current analysis is based on women's data recruited from 20 districts in West Bengal. The dataset comprises 20,925 women from West Bengal, representing a small fraction of the 7,24,115 women in India. The study focused exclusively on women from the SC and ST social categories in West Bengal, resulting in the inclusion of 7,706 women in these groups from the sample of 20,925. Pregnant women were excluded from the study due to limitations in accurately calculating BMI during pregnancy. 249 were pregnant at the time of the survey and were therefore not included in the analysis. The study's final sample size is 7,457 women, consisting of 5,961 women from the SC category and 1,496 women from the ST category.

### Variables description

#### Outcome variable

Body Mass Index (BMI) was taken as the Outcome variable. We are taking only the non-pregnant women in the reproductive age group, and BMI are categorized into 2 groups underweight and non-underweight. Less than 18.5 kg/${m}^{2}$ coded as 0 “underweight”, 18.5 and more kg/${m}^{2}$ coded as 1 “non-underweight”.

#### Explanatory variables

A binary logistic regression model was employed to determine the impact of various independent variables on the study participants' nutritional status (BMI). The independent variables were included different socio-economic and bio-demographic factors such as caste, religion, place of residence, wealth index, age group, level of education, marital status, parity, and family size. Furthermore, food and dietary habits such as milk or curd, pulses or beans, dark green leafy vegetables, fruits, eggs, fish, chicken or meat, fried foods, aerated drinks; alcohol and smoking behaviors and geographic region were also considered as independent variables in the analysis. The aim was to examine how these background characteristics or variables impact the nutritional status of the study participants.

### Statistical analysis

Multivariate decomposition analysis is a powerful tool that can be used to identify the factors that contribute to a specific outcome or phenomenon. The analysis provides valuable insights into the factors that must be addressed to reduce the nutritional gap between SC and ST reproductive-aged women in West Bengal. STATA software was used to do the complete data analysis. The study encompassed all 20 districts of West Bengal to investigate the geographical variance of nutritional status among SC and ST women. Spatial tool, QGIS 2.18.25 software, was used to show the distribution of underweight, and non-underweight SC and ST women in West Bengal.

Multivariate decomposition analysis was used to identify the contributions of covariates that explain the group differences to average predictions. The decomposition analysis aimed to identify covariates that contributed to the difference in nutritional status (BMI) by SC and ST respondents. The multivariate decomposition analysis has two contribution effects: compositional differences (endowments) ‘E’ and the effects of characteristics that differ in the coefficients or behavioural change ‘C’ responses for the selected predictor variables. The observed differences in nutritional status (BMI) thus can be additively decomposed into a characteristic (or endowment) component and a coefficient (or effects of characteristics) component. In the non-linear model, the dependent variable is a function of a linear combination of predictors and regression coefficients:

$$\mathrm{Y }=\mathrm{ F}(\mathrm{X\beta }) =\mathrm{ e}(\mathrm{X\beta })/(1+\mathrm{ e}(\mathrm{X\beta }))$$

where Y denotes the n*1 dependent variable vector, X an n*K matrix of independent variables and β a K*1 vector of coefficients. The proportion difference in Y between SC A and ST B of nutritional status (BMI) can be decomposed as:

$${\mathrm{Y}}_{\mathrm{A}} - {\mathrm{Y}}_{\mathrm{B}} =\mathrm{ F}({\mathrm{X}}_{\mathrm{A}}{\upbeta }_{\mathrm{A}}) -\mathrm{ F}({\mathrm{X}}_{\mathrm{B}}{\upbeta }_{\mathrm{B}})$$

For the log odds of nutritional status (BMI), the proportion of the model is written as.

$$\begin{array}{ccc}\mathrm{Logit}({\mathrm Y}_{\mathrm A})-\mathrm{Logit}({\mathrm Y}_{\mathrm B})&=&\mathrm F({\mathrm X}_{\mathrm A}{\mathrm\beta}_{\mathrm A})-\mathrm F({\mathrm X}_{\mathrm B}{\mathrm\beta}_{\mathrm B})\\\underbrace{=\mathrm F({\mathrm X}_{\mathrm A}{\mathrm\beta}_{\mathrm A})-\mathrm F({\mathrm X}_{\mathrm B}{\mathrm\beta}_{\mathrm A})}_{\mathrm E}&+&\underbrace{\mathrm F({\mathrm X}_{\mathrm B}{\mathrm\beta}_{\mathrm A})-\mathrm F({\mathrm X}_{\mathrm B}{\mathrm\beta}_{\mathrm B})}_{\mathrm C}\end{array}$$

The component ‘E’ is the difference attributable to endowment change, usually called the explained component. The ‘C’ component is the difference attributable to coefficient (behavioural) change, usually called the unexplained component. The model structure for the decomposition analysis was:

$$\mathrm{Logit}\left(\mathrm{A}\right)-\mathrm{Logit}\left(\mathrm{B}\right)= \left[{\upbeta }_{0\mathrm{A}}-{\upbeta }_{0\mathrm{B}}\right]+ \sum {\upbeta }_{\mathrm{ijA}}\left[{\mathrm{X}}_{\mathrm{ijA}}-{\mathrm{X}}_{\mathrm{ijB}}\right]+ \sum {\mathrm{X}}_{\mathrm{ijB}}\left[{\upbeta }_{\mathrm{ijA}}-{\upbeta }_{\mathrm{ijB}}\right],$$

where

- β_0A_ is the intercept in the regression equation for SC
- β_0B_ is the intercept in the regression equation for ST
- β_ijA_ is the coefficient of the j^th^ category of the i^th^ determinant for SC
- β_ijB_ is the coefficient of j^th^ category of the i^th^ determinant for ST
- X_ijA_ is the proportion of the j^th^ category of the i^th^ determinant for SC
- X_ijB_ is the proportion of the j^th^ category of the i^th^ determinant for ST

The command mvdcmp was used to perform multivariate decomposition analysis in STATA 16.

## Result

The study investigated the nutritional status and differential background characteristics among SC and ST women in West Bengal, India. BMI was used as an essential health indicator to determine nutritional status. Table 1 shows the different background characteristics and the BMI distribution among SC and ST women in West Bengal. The results showed that undernutrition affects the ST population more than the SC population, as 23.63% of ST women were underweight compared to 15.72% of SC women. Over half of all Scheduled women belonged to the non-underweight BMI category. Age was a significant factor affecting nutritional status, as the age group 15–24 had the highest prevalence of underweight, while the age group 35 and above had the highest prevalence of not underweight. In the 15–24 year age group, ST women (31.06%) were more underweight than SC women (26.35%), whereas, in the 25–34 and 35–49 age groups, SC women (87.32% and 90.64%) were found to be more non-underweight than ST women (81.23% and 82.03%), respectively. Marital status was also a significant factor affecting nutritional status, as both SC and ST unmarried women (29.84% and 26.62%, respectively) were more underweight than married women (12.90% and 21.35%, respectively). Religion and place of residence were also significant factors affecting nutritional status. Regarding religion, SC Hindu women were found to have 84.28% non-underweight, while other religious SC women had 8.62% underweight. In terms of place of residence, rural women (23.96%) were more underweight than urban women (10.27%). Educational status and wealth quintile were found to impact nutritional status. Higher-educated women were more non-underweight than those with no education. Highly-educated SC women were found to be more underweight (9.45%) than highly educated ST women (3.66%). The poorest SC women, 24.05%, were underweight compared to 75.95% who were non-underweight. Similarly, among the poorest ST women, 25.97% were underweight compared to 74.03% who were non-underweight. However, as the wealth quintile increased, the percentage of underweight women decreased. Among the poorer SC women, 14.29% were underweight compared to 85.71% who were non-underweight. Among the richer SC women, only 2.64% were underweight compared to 97.36% who were non-underweight. Similarly, among the richer ST women, only 10.20% were underweight compared to 89.80% who were non-underweight.

**Table 1:** Percentage distribution of SC & ST women by underweight & non-underweight BMI, West Bengal, NFHS-5 (2019-21)

	**Background Characteristics**	**Scheduled Caste Women**			**Scheduled Tribe Women**		
		**Non-underweight**	**Underweight**	**Sample Size**	**Non-underweight**	**Underweight**	**Sample Size**
**Socio-demographic factors**	**Religion**						
	Hindu	84.28	15.72	5,880	76.37	23.63	1,291
	Non Hindu	91.38	8.62	81	83.31	16.69	205
	**Place of Residence**						
	Urban	90.85	9.15	1,599	89.73	10.27	119
	Rural	81.60	18.40	4,362	76.04	23.96	1,377
	**Wealth Index**						
	Poorest	75.95	24.05	2,322	74.03	25.97	1,008
	Poorer	85.71	14.29	1,777	78.62	21.38	303
	Middle	91.19	8.81	1,119	91.34	8.66	112
	Richer	92.69	7.31	566	94.43	5.57	63
	Richest	97.36	2.64	177	89.80	10.20	10
	**Age Group**						
	15–24	73.65	26.35	1,858.00	68.94	31.06	516
	25–34	87.32	12.68	1,760.00	81.23	18.77	441
	35–49	90.64	9.36	2,343.00	82.03	17.97	539
	**Education**						
	No Education	83.33	16.67	1,435	77.11	22.89	581
	Primary	87.69	12.31	1,137	78.07	21.93	255
	Secondary	82.71	17.29	2,975	75.02	24.98	598
	Higher	90.55	9.45	414	96.34	3.66	62
	**Marital Status**						
	Unmarried	70.16	29.84	999	73.38	26.62	325
	Married	87.10	12.90	4654	78.65	21.35	1,062
	Others	88.27	11.73	308	75.57	24.43	109
	**Parity**						
	0	72.81	27.19	1,334	74.12	25.88	461
	1 to 2	88.02	11.98	3,273	78.53	21.47	668
	2 & more	86.96	13.04	1,354	78.91	21.09	367
	**Family Size**						
	< 4	88.14	11.86	1,489	79.05	20.95	350
	4 & More	83.08	16.92	4,472	76.76	23.24	1,146
**Food & dietary habits**	**Milk or curd**						
	Never	78.58	21.42	868	76.03	23.97	370
	Daily	84.97	15.03	1,196	76.11	23.89	205
	Weekly	87.09	12.91	1,656	82.23	17.77	317
	Occasionally	84.04	15.96	2,241	75.67	24.33	604
	**Pulses or beans**						
	Never	81.67	18.33	92	73.80	26.20	18
	Daily	85.51	14.49	2813	76.05	23.95	630
	Weekly	83.89	16.11	2677	78.29	21.71	720
	Occasionally	80.17	19.83	379	78.15	21.85	128
	**Dark green leafy vegetables**						
	Daily	84.60	15.40	4,599	79.00	21.00	1,142
	Weekly	83.87	16.13	1,188	72.71	27.29	317
	Occasionally	82.44	17.56	174	70.16	29.84	37
	**Fruits**						
	Never	80.27	19.73	315	75.61	24.39	139
	Daily	87.47	12.53	335	88.57	11.43	63
	Weekly	88.11	11.89	1,766	78.09	21.91	309
	Occasionally	82.36	17.64	3,545	76.69	23.31	985
	**Eggs**						
	Never	83.93	16.07	242	72.25	27.75	79
	Daily	86.58	13.42	618	69.73	30.27	93
	Weekly	84.46	15.54	4,181	77.86	22.14	1,040
	Occasionally	82.53	17.47	920	79.40	20.60	284
	**Fish**						
	Never	82.51	17.49	82	80.70	19.30	33
	Daily	86.48	13.52	1,296	80.54	19.46	170
	Weekly	84.13	15.87	3,835	78.01	21.99	1,010
	Occasionally	81.55	18.45	748	72.01	27.99	283
	**Chicken or meat**						
	Never	89.62	10.38	238	79.58	20.42	28
	Daily	89.36	10.64	85	83.01	16.99	30
	Weekly	85.28	14.72	3,216	77.44	22.56	800
	Occasionally	82.40	17.60	2,422	76.76	23.24	638
	**Fried foods**						
	Never	86.20	13.80	207	76.98	23.02	56
	Daily	85.06	14.94	1,100	78.02	21.98	234
	Weekly	83.49	16.51	2,612	78.36	21.64	586
	Occasionally	84.94	15.06	2,042	75.98	24.02	620
	**Aerated drinks**						
	Never	81.08	18.92	1,396	76.05	23.95	507
	Daily	88.83	11.17	96	94.33	5.67	12
	Weekly	86.45	13.55	532	76.47	23.53	95
	Occasionally	85.05	14.95	3,937	77.93	22.07	882
**Alcohol & smoking habits**	**Smoke**						
	No	84.38	15.62	5,953	77.39	22.61	1,492
	Yes	88.72	11.28	8	38.58	61.42	4
	**Drink Alcohol**						
	No	84.47	15.53	5,911	77.31	22.69	1,420
	Yes	71.77	28.23	50	77.03	22.97	76
**Geographic factor**	**Region**						
	North Bengal	84.50	15.50	1,542	76.74	23.26	455
	Middle Bengal	79.36	20.64	1,280	74.87	25.13	330
	South Bengal	85.61	14.39	3,139	78.34	21.66	711
	**Total**	84.38	15.62	5,961	76.14	23.86	1,496

Food and dietary habits were also significant factors affecting nutritional status. The results show that consumption of milk or curd is associated with lower rates of underweight among both SC and ST women. Those who had never consumed milk or curd had higher rates of underweight, with 21.42% of SC and 23.97% of ST women falling under this category. Similarly, for pulses or beans, occasional intake was associated with higher rates of underweight among SC women (19.83%). In comparison, ST women who had never consumed pulses or beans had the highest rates of underweight (26.20%). Daily consumption of dark green leafy vegetables was associated with lower rates of underweight among both SC and ST women, with 15.40% and 21.00%, respectively, falling under the underweight category. In contrast, women who had never consumed fruits had higher rates of underweight, with 19.73% of SC and 24.39% of ST women being underweight. For eggs, 16.02% of underweight SC women and 24.75% of underweight ST women had never consumed them. Likewise, 17.49% of underweight SC women and 19.30% of underweight ST women had never consumed fish. For ST women, occasional fish intake was associated with higher rates of underweight (27.99%). Among both SC and ST women, occasional chicken and meat intake was associated with higher rates of underweight (17.60% and 23.24% respectively). Weekly intake of fried foods was associated with higher rates of underweight among SC women (16.51%), while occasional intake of fried foods was associated with higher rates of underweight among ST women (24.02%). Finally, among both SC and ST women, those who never consumed aerated drinks had higher rates of underweight (18.92% and 23.95% respectively).

In terms of alcohol and smoking habits, a high percentage of ST women had a habit of smoking (61.42%) compared to SC women (11.28%). Similarly, a higher percentage of SC women (28.23%) had a habit of drinking alcohol compared to ST women (22.97%).

Finally, the study also found regional differences in nutritional status, with Middle Bengal having a higher percentage of underweight women among both SC and ST populations.

**Fig. 1:**
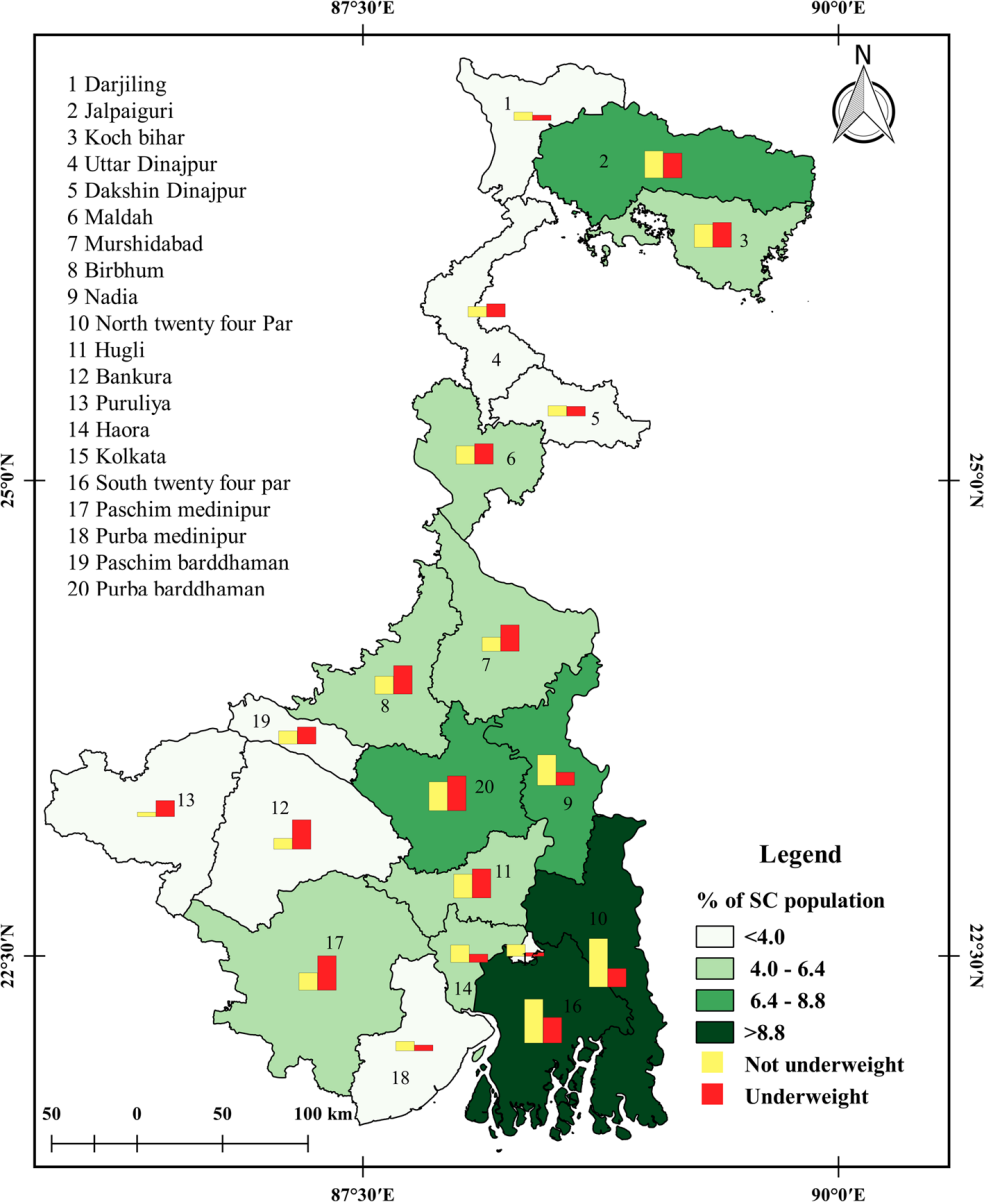
Distribution of underweight and non-underweight women of reproductive age in SC communities across districts of West Bengal, NFHS-5 (2019–21)

**Fig. 2:**
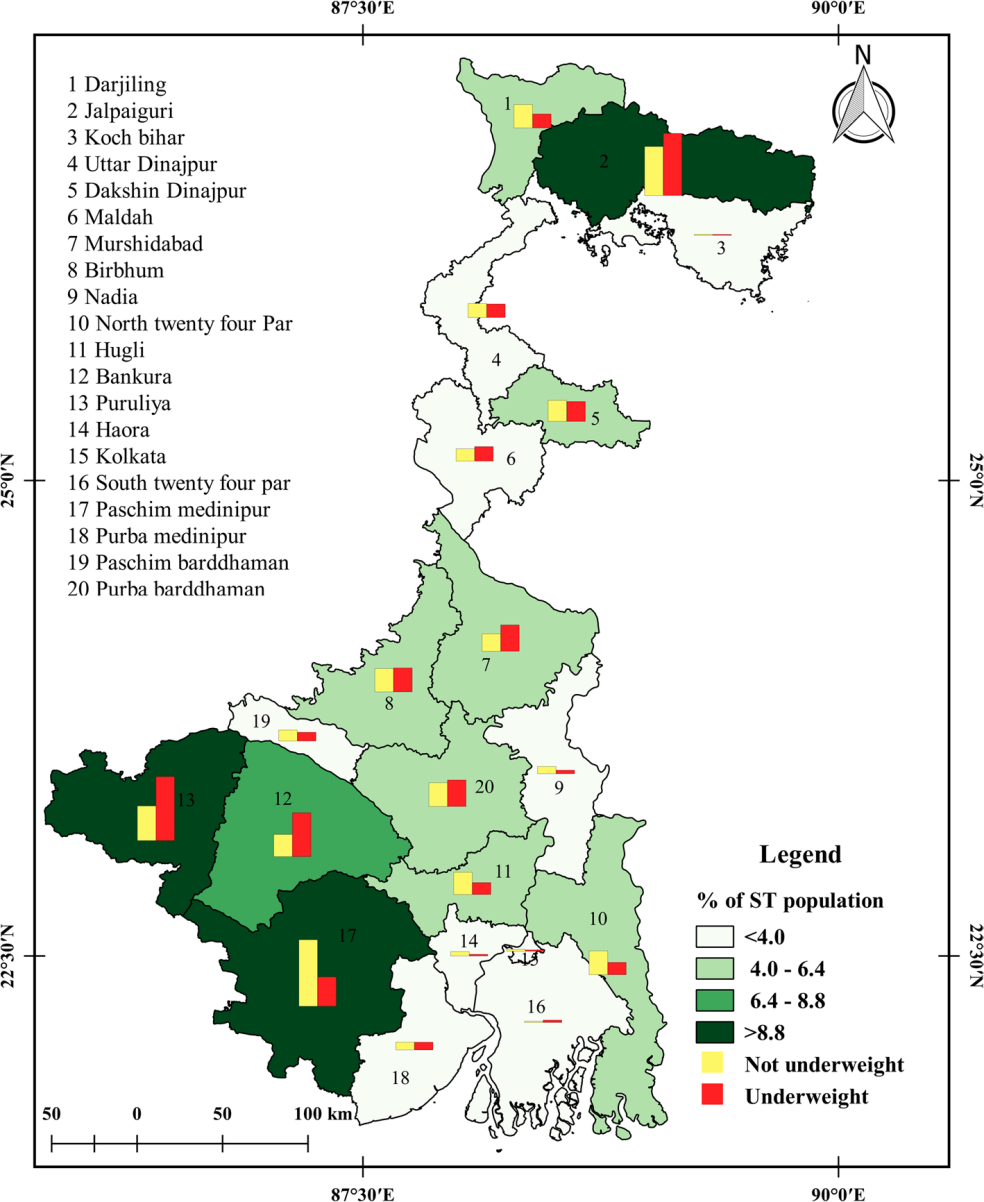
Distribution of underweight and non-underweight women of reproductive age in ST communities across districts of West Bengal, NFHS-5 (2019–21)

The distribution of underweight and non-underweight SC and ST reproductive-aged women across different districts of West Bengal is shown in Figs. 1 and 2. The figure shows that some districts in the southern region of West Bengal have a higher percentage of underweight SC women compared to the northern districts. Purba Barddhaman (8.95%), Paschim Medinipur (8.84%), and Bankura (7.54%) are some of the districts where underweight SC women were found in a higher percentage. In contrast, Kolkata (0.99%), Darjeeling (1.36%), and Purba Medinipur (1.49%) are some of the districts where underweight SC women were found in a low percentage. The results show a clear regional variation in the nutritional status of SC women in West Bengal.

Similarly, the figure shows that Puruliya (16.41%), Jalpaiguri (15.93%), and Bankura (11.27%) are some of the districts with higher percentages of underweight ST women. Cooch Behar (0.22%), Kolkata (0.37%), Howrah (0.44%), and South 24 Parganas (0.62%) are some of the districts where the percentage of underweight ST women was low. However, the results for ST women were different from SC women, as no clear regional patterns were observed for the distribution of underweight ST women across districts of West Bengal. In most districts, the underweight percentage among ST women ranged from 0.93% to 7.56%. The highest percentage of non-underweight ST reproductive-aged women was found in Paschim Medinipur (17.03%), Jalpaiguri (12.57%), Puruliya (8.91%), and North 24 Paraganas (6.12%).

It is worth noting that Cooch Behar, Kolkata, and South 24 Parganas had very low percentages of ST residents, according to the census of India in 2011. The findings of this study highlight the importance of understanding the regional variation in nutritional status among different social groups and the need for targeted interventions to improve the nutritional status of women in West Bengal.

This study aimed to determine the odds of underweight and non-underweight reproductive-aged SC and ST women in West Bengal and identify the socio-demographic, dietary and lifestyle factors associated with their nutritional status. Binary logistic regression analysis shown in Table 2 revealed significant associations between socio-demographic factors and underweight status among SC and ST women in West Bengal.

**Table 2:** Odds of reproductive-aged SC & ST women by underweight & not underweight BMI, West Bengal, NFHS-5 (2019-21): Result from binary logistic regression analysis

	**Background Characteristics**	**Scheduled Caste**	**Scheduled Tribe**	**Combined Model**
**Socio-demographic factors**	**Caste**			
	Scheduled Caste ®			1[1.00,1.00]
	Scheduled Tribe			1.02^c^ [0.90,1.12]
	**Religion**			
	Non Hindu ®	1[1.00,1.00]	1[1.00,1.00]	1[1.00,1.00]
	Hindu	2.75^a^ [1.69,4.47]	1.31[0.97,1.77]	1.61^a^ [1.26,2.05]
	**Place of Residence**			
	Urban ®	1[1.00,1.00]	1[1.00,1.00]	1[1.00,1.00]
	Rural	1.38^a^ [1.21,1.57]	1.34[0.82,2.19]	1.35^a^ [1.19,1.54]
	**Wealth Index**			
	Poorest ®	1[1.00,1.00]	1[1.00,1.00]	1[1.00,1.00]
	Poorer	0.59^a^ [0.53,0.66]	0.8[0.63,1.02]	0.63^a^ [0.57,0.69]
	Middle	0.38^a^ [0.33,0.45]	0.32^a^ [0.19,0.54]	0.39^a^ [0.34,0.45]
	Richer	0.36^a^ [0.29,0.46]	0.30^b^ [0.13,0.68]	0.37^a^ [0.30,0.46]
	Richest	0.16^a^ [0.09,0.27]	0.56[0.12,2.65]	0.17^a^ [0.10,0.28]
	**Age Group**			
	15–24 ®	1[1.00,1.00]	1[1.00,1.00]	1[1.00,1.00]
	25–34	0.49^a^ [0.43,0.56]	0.42^a^ [0.32,0.55]	0.47^a^ [0.42,0.53]
	35–49	0.31^a^ [0.27,0.37]	0.33^a^ [0.24,0.46]	0.31^a^ [0.27,0.36]
	**Education**			
	No Education ®	1[1.00,1.00]	1[1.00,1.00]	1[1.00,1.00]
	Primary	0.71^a^ [0.61,0.82]	0.76^c^ [0.58,0.99]	0.70^a^ [0.62,0.80]
	Secondary	0.63^a^ [0.54,0.73]	0.71^c^ [0.54,0.93]	0.63^a^ [0.55,0.71]
	Higher	0.37^a^ [0.28,0.48]	0.12^a^ [0.05,0.30]	0.33^a^ [0.26,0.43]
	**Marital Status**			
	Unmarried ®	1[1.00,1.00]	1[1.00,1.00]	1[1.00,1.00]
	Married	0.66^a^ [0.55,0.80]	0.88[0.62,1.25]	0.69^a^ [0.58,0.81]
	Others	0.67^b^ [0.50,0.90]	1.14[0.71,1.84]	0.78[0.61,1.00]
	**Parity**			
	0 ®	1[1.00,1.00]	1[1.00,1.00]	1[1.00,1.00]
	1 to 2	0.66^a^ [0.55,0.80]	1.1[0.79,1.53]	0.76^a^ [0.65,0.89]
	2 & more	0.72^b^ [0.57,0.90]	1.2[0.82,1.75]	0.83^c^ [0.68,1.00]
	**Family Size**			
	< 4 ®	1[1.00,1.00]	1[1.00,1.00]	1[1.00,1.00]
	4 & More	1.27^a^ [1.13,1.43]	0.99[0.79,1.24]	1.20^a^ [1.08,1.32]
**Food & dietary habits**	**Milk or curd**			
	Never ®	1[1.00,1.00]	1[1.00,1.00]	1[1.00,1.00]
	Daily	0.80^b^ [0.68,0.94]	1.11[0.81,1.53]	0.88[0.76,1.01]
	Weekly	0.69^a^ [0.60,0.80]	0.70^c^ [0.53,0.94]	0.72^a^ [0.64,0.82]
	Occasionally	0.76^a^ [0.66,0.87]	1.16[0.92,1.45]	0.86^b^ [0.76,0.96]
	**Pulses or beans**			
	Never ®	1[1.00,1.00]	1[1.00,1.00]	1[1.00,1.00]
	Daily	1[0.70,1.42]	1.22[0.57,2.65]	1.03[0.75,1.41]
	Weekly	0.96[0.67,1.36]	0.89[0.41,1.91]	0.93[0.68,1.27]
	Occasionally	1.1[0.75,1.62]	0.74[0.33,1.67]	0.96[0.68,1.35]
	**Dark green leafy vegetables**			
	Daily ®	1[1.00,1.00]	1[1.00,1.00]	1[1.00,1.00]
	Weekly	0.96[0.86,1.08]	1.64^a^ [1.33,2.03]	1.1[1.00,1.22]
	Occasionally	0.79[0.60,1.03]	1.46[0.87,2.45]	0.91[0.72,1.15]
	**Fruits**			
	Never ®	1[1.00,1.00]	1[1.00,1.00]	1[1.00,1.00]
	Daily	1.48^b^ [1.10,2.00]	0.67[0.35,1.32]	1.31^c^ [1.01,1.71]
	Weekly	0.99[0.79,1.24]	1.03[0.72,1.48]	1[0.83,1.21]
	Occasionally	1.14[0.93,1.40]	0.91[0.68,1.22]	1.09[0.92,1.29]
	**Eggs**			
	Never ®	1[1.00,1.00]	1[1.00,1.00]	1[1.00,1.00]
	Daily	0.70^c^ [0.52,0.95]	1.09[0.67,1.79]	0.76^c^ [0.59,0.97]
	Weekly	0.84[0.65,1.10]	0.57^b^ [0.39,0.84]	0.78^c^ [0.63,0.97]
	Occasionally	0.84[0.63,1.11]	0.43^a^ [0.28,0.66]	0.73^b^ [0.58,0.92]
	**Fish**			
	Never ®	1[1.00,1.00]	1[1.00,1.00]	1[1.00,1.00]
	Daily	0.74[0.48,1.15]	0.95[0.46,1.95]	0.8[0.56,1.16]
	Weekly	0.85[0.55,1.31]	1.07[0.54,2.13]	0.92[0.64,1.31]
	Occasionally	0.9[0.57,1.41]	1.56[0.77,3.18]	1.05[0.72,1.52]
	**Chicken or meat**			
	Never ®	1[1.00,1.00]	1[1.00,1.00]	1[1.00,1.00]
	Daily	1.12[0.66,1.92]	1.11[0.42,2.88]	1.17[0.74,1.85]
	Weekly	1.38^c^ [1.00,1.91]	1.67[0.90,3.10]	1.44^c^ [1.09,1.91]
	Occasionally	1.39^c^ [1.01,1.92]	1.51[0.81,2.82]	1.42^c^ [1.07,1.88]
	**Fried foods**			
	Never ®	1[1.00,1.00]	1[1.00,1.00]	1[1.00,1.00]
	Daily	1.21[0.91,1.61]	1.13[0.68,1.88]	1.19[0.94,1.52]
	Weekly	1.26[0.96,1.66]	0.98[0.61,1.57]	1.17[0.93,1.48]
	Occasionally	1.17[0.89,1.54]	1.06[0.67,1.69]	1.12[0.89,1.42]
	**Aerated drinks**			
	Never ®	1[1.00,1.00]	1[1.00,1.00]	1[1.00,1.00]
	Daily	0.73[0.47,1.12]	0.21[0.03,1.45]	0.68[0.45,1.03]
	Weekly	0.89[0.74,1.08]	1.08[0.72,1.60]	0.92[0.78,1.09]
	Occasionally	0.9[0.80,1.01]	0.94[0.77,1.15]	0.92[0.83,1.01]
**Alcohol & smoking habits**	**Smoke**			
	No ®	1[1.00,1.00]	1[1.00,1.00]	1[1.00,1.00]
	Yes	0.84[0.14,4.99]	8.60^c^ [1.56,47.33]	2.65[0.96,7.29]
	**Drink Alcohol**			
	No ®	1[1.00,1.00]	1[1.00,1.00]	1[1.00,1.00]
	Yes	2.12^b^ [1.33,3.37]	1.06[0.70,1.61]	1.41^c^ [1.04,1.90]
**Geographic factor**	**Region**			
	North Bengal ®	1[1.00,1.00]	1[1.00,1.00]	1[1.00,1.00]
	Middle Bengal	1.22^b^ [1.05,1.42]	0.82[0.62,1.07]	1.12[0.98,1.27]
	South Bengal	1[0.88,1.13]	0.74^c^ [0.58,0.94]	0.93[0.84,1.04]
	**Observation**	5,961	1496	7457

^a^Significant at 1%, ^b^Significant at 5%, ^c^Significant at 10%

The odds of being underweight were 2.75 times higher (95% CI: 1.69, 4.47) among Hindu average BMI women compared to other religious women in SC group. The probability of being underweight was significantly 1.61 times more (95% CI: 1.26, 2.05) among SC and ST Hindu women combinedly compared to women of non-hindu religions. Rural SC and ST women had 1.38 times higher (95% CI: 1.21, 1.57) odds of being underweight compared to urban SC and ST women.

Moreover, increasing wealth index was associated with a lower likelihood of underweight among SC and ST women. Middle and richer SC women had 62% and 64% less likely chances of being underweight respectively, while the corresponding figures for ST women were 68% and 70%. Increasing age was also associated with a lower likelihood of being underweight among SC and ST women, with women aged 25–34 and 35–49 having 53% and 69% less probability of underweight than women aged 15–24. Education was also correlated with underweight status among SC and ST women, with higher-educated women having lower odds of being underweight compared to uneducated women. The odds of being underweight were 63% and 88% less (95% CI: 0.09, 0.50) among higher-educated SC and ST women, respectively, compared to uneducated women. Married SC women had 34% lower odds of being underweight compared to unmarried SC women.

Food and dietary habits were associated with underweight status among SC and ST women in West Bengal. SC women who consumed milk or curd daily, weekly and occasionally had 20%, 31% and 24% lower odds of being underweight, respectively, than those who had never consumed milk or curd. Similarly, ST women who consumed eggs weekly and occasionally had 43% and 57% lower odds of being underweight than those who had never consumed eggs. Alcohol intake was also significantly associated with underweight status among SC women.

Geographic factors were associated with underweight status among SC women in West Bengal. Although the region showed insignificant relationship with nutrition status for South Bengal compared to North Bengal, for Middle Bengal, SC women had 1.22 times (95% CI: 1.05, 1.42) higher odds of being underweight compared to North Bengal.

Overall, this study highlights the need for targeted interventions to improve the nutritional status of marginalized communities like SC and ST women in West Bengal, considering their socio-demographic, dietary, and lifestyle factors.

The results of the decomposition analysis are presented in Table 3. The table shows the percentage contribution of each factor to the nutritional gap between the SC and ST reproductive-aged women population in West Bengal.

**Table 3:** Multivariate logistic regression decomposition estimates for SC-ST differentials in underweight & not underweight BMI among reproductive aged women, West Bengal, NFHS-5 (2019-21)

	**Background characteristics**	**Due to difference in characteristics**					**Due to the difference in coefficients**				
		**Coef**	**SE**	***p*** **-value**	**% Contribution**		**Coef**	**SE**	***p*** **-value**	**% Contribution**	
**Socio-demographic factors**	**Wealth Index**										
	Poorest	0.000	0.000			43.54	0.000	0.000			2.1
	Poorer	0.005	0.002	0.044	6.68		0.009	0.026	0.729	13.26	
	Middle	0.017	0.005	0.001	24.24		-0.015	0.048	0.758	-21.53	
	Richer	0.008	0.004	0.056	11.15		-0.001	0.015	0.943	-1.52	
	Richest	0.001	0.003	0.771	1.47		0.008	0.022	0.712	11.89	
	**Age Group**										
	15–24	0.000	0.000			5.89	0.000	0.000			14.38
	25–34	0.000	0.000	0.000	0.07		-0.005	0.021	0.815	-7.32	
	35–49	0.004	0.001	0.000	5.82		0.014	0.042	0.722	21.7	
	**Place of Residents**										
	Urban	0.000	0.000			15.44	0.000	0.000			43.56
	Rural	0.011	0.009	0.226	15.44		0.030	0.118	0.799	43.56	
	**Education**										
	No education	0.000	0.000			16.51	0.000	0.000			-10.28
	Primary	0.001	0.001	0.297	0.77		0.001	0.010	0.899	1.90	
	Secondary	0.004	0.003	0.11	5.96		0.007	0.031	0.817	10.33	
	Higher	0.007	0.002	0.002	9.78		-0.016	0.046	0.734	-22.51	
	**Marital Status**										
	Never Married	0.000	0.000			0.88	0.000	0.000			128.8
	Married	0.000	0.002	0.980	0.08		0.083	0.225	0.712	120.58	
	Others	0.001	0.001	0.568	0.80		0.006	0.016	0.721	8.22	
	**Parity**										
	0	0.000	0.000			0.14	0.000	0.000			77.58
	1 to 2	0.000	0.003	0.999	0.01		0.043	0.117	0.712	62.54	
	More than 2	0.000	0.001	0.892	0.13		0.010	0.032	0.744	15.04	
	**Family Size**										
	< 4	0.000	0.000			0.34	0.000	0.000			-43.51
	4 & More	0.000	0.000	0.479	0.34		-0.030	0.084	0.722	-43.51	
**Food & dietary habits**	**Milk or curd**										
	Never	0.000	0.000			0.79	0.023	0.060	0.708	32.84	76.22
	Daily	0.000	0.000	0.164	-0.29		0.010	0.030	0.734	14.53	
	Weekly	0.001	0.001	0.511	1.08		0.042	0.114	0.709	61.69	
	Occasionally										
	**Pulses or beans**										
	Never	0.000	0.000			-4.69	0.000	0.000			40.48
	Daily	-0.002	0.004	0.638	-2.52		0.030	0.106	0.778	43.56	
	Weekly	0.000	0.002	0.958	-0.18		0.003	0.065	0.963	4.43	
	Occasionally	-0.001	0.002	0.423	-1.99		-0.005	0.017	0.759	-7.51	
	**Dark green leafy vegetables**										
	Daily	0.000	0.000			0.71	0.000	0.000			35.04
	Weekly	0.001	0.000	0.011	0.97		0.021	0.056	0.712	29.95	
	Occasionally	0.000	0.000	0.425	-0.26		0.004	0.010	0.719	5.09	
	**Fruits**										
	Never	0.000	0.000			3.80	0.000	0.000			-91.98
	Daily	0.001	0.001	0.096	2.16		-0.013	0.036	0.710	-19.45	
	Weekly	0.003	0.003	0.288	4.85		-0.011	0.036	0.769	-15.51	
	Occasionally	-0.002	0.002	0.229	-3.21		-0.039	0.111	0.723	-57.02	
	**Eggs**										
	Never	0.000	0.000			-4.93	0.000	0.000			-103.6
	Daily	0.000	0.002	0.982	0.06		0.007	0.022	0.743	10.38	
	Weekly	0.000	0.000	0.043	0.67		-0.053	0.154	0.728	-77.42	
	Occasionally	-0.004	0.001	0.007	-5.66		-0.025	0.069	0.714	-36.56	
	**Fish**										
	Never	0.000	0.000			6.26	0.000	0.000			34.07
	Daily	0.001	0.007	0.863	1.71		0.004	0.032	0.897	6.02	
	Weekly	0.000	0.002	0.961	0.14		0.011	0.090	0.906	15.46	
	Occasionally	0.003	0.004	0.455	4.41		0.009	0.029	0.761	12.59	
	**Chicken or meat**										
	Never	0.000	0.000			0.58	0.000	0.000			71.45
	Daily	0.000	0.001	0.976	-0.02		0.001	0.003	0.844	0.98	
	Weekly	0.000	0.000	0.501	-0.29		0.034	0.117	0.770	49.5	
	Occasionally	0.001	0.001	0.628	0.89		0.014	0.066	0.825	20.97	
	**Fried foods**										
	Never	0.000	0.000			1.07	0.000	0.000			-80.49
	Daily	0.000	0.001	0.779	0.55		-0.011	0.034	0.747	-15.86	
	Weekly	0.001	0.002	0.667	1.29		-0.032	0.095	0.733	-46.79	
	Occasionally	-0.001	0.003	0.866	-0.77		-0.012	0.045	0.784	-17.84	
	**Aerated drinks**										
	Never	0.000	0.000			1.67	0.000	0.000			6.83
	Daily	0.001	0.001	0.332	1.59		-0.002	0.008	0.770	-3.30	
	Weekly	-0.001	0.001	0.493	-0.97		0.008	0.022	0.718	11.41	
	Occasionally	0.001	0.001	0.591	1.05		-0.001	0.027	0.973	-1.28	
**Alcohol & smoking habits**	**Smoke**										
	No	0.000	0.000			0.41	0.000	0.000			0.84
	Yes	0.000	0.000	0.167	0.41		0.001	0.002	0.724	0.84	
	**Drink Alcohol**										
	No	0.000	0.000			0.04	0.000	0.000			-2.16
	Yes	0.000	0.002	0.985	0.04		-0.001	0.004	0.708	-2.16	
**Geographic factor**	**Region**										
	North Bengal	0.000	0.000			0.79	0.000	0.000			-70.18
	Middle Bengal	0.000	0.000	0.164	-0.29		-0.019	0.053	0.718	-27.61	
	South Bengal	0.001	0.001	0.511	1.08		-0.029	0.083	0.724	-42.57	

The results indicate that socio-demographic factors were the major contributors to the gap. Wealth index was found to be the most significant factor, contributing to 43.54% of the gap, followed by education (16.51%), place of residence (15.44%), and age group (5.89%). These findings suggest that addressing socio-economic disparities and improving access to education could help reduce the nutritional gap between SC and ST women.

Food and dietary factors were also found to contribute significantly to the gap. Fish was the most significant dietary factor, contributing to 6.26% of the gap, followed by fruits (3.81%), aerated drinks (1.67%), and fried foods (1.07%). These findings highlight the importance of promoting a healthy and balanced diet to reduce the nutritional gap between SC and ST women.

Smoking behaviour, alcohol consumption, and geographic differences contributed relatively small percentages to the gap, indicating that they may have a limited impact on the nutritional status of SC and ST women in West Bengal.

Marital statuses, parity, consumption of milk or curd, green vegetables, and chicken or meat were found to have minimal contributions to the gap. In contrast, the consumption of pulses or beans and eggs was an important factor that helped decrease the gap between SC and ST women. These findings suggest that promoting the consumption of pulses or beans and eggs could be an effective strategy to improve the nutritional status of SC and ST women in West Bengal.

Overall, the findings of the decomposition analysis provide essential insights into the factors contributing to the nutritional gap between SC and ST reproductive-aged women population in West Bengal. The results highlight the need for targeted interventions that address socio-economic disparities and promote a healthy and balanced diet to reduce the gap and improve the population's nutritional status.

## Discussion

The present study aimed to investigate the nutritional status of SC and ST women in West Bengal by analyzing data from the National Family Health Survey-5. The findings of the decomposition analysis revealed that socio-demographic factors, especially wealth index, education, place of residence, and age group were the major contributors to the nutritional gap between SC and ST women. Food and dietary factors, such as fish, fruits, aerated drinks, and fried foods, also significantly contributed to the gap.

The results of the present study are consistent with the findings of earlier studies that have investigated the nutritional status of SC and ST women in India. For instance, Biswas et al. (2022), Haregu et al. (2018), Nie (2021), and Rout (2009) have highlighted the impact of socio-demographic factors such as religion, place of residence, wealth index, age group, education, marital status, parity, and family size on the nutritional status of women in India [22–25]. Similarly, Kanguru et al. (2017) have identified cultural and social values regarding diet and care during and after pregnancy as important factors that contribute to weight gain in women [26].

Women from SCs and STs who come from a poor economic backgrounds are more likely to be underweight than women from other economic backgrounds. A woman's wealth status influences her likelihood of being overweight or obese, according to a comparable study conducted in 54 developing and underdeveloped countries among women of reproductive age groups. The overall picture reveals poor nutritional status among Scheduled women, particularly ST women, who are more vulnerable [27].

We used education as a SES indicator in our analysis. In general, those with higher education levels are less likely to be underweight. This is explained by the fact that educated people have more knowledge of nutrition and health. On the other hand, poor levels of education may cause underweight as a result of indifference. Education is a more consistent measure of SES throughout one's life than career and wealth, and it might reflect early circumstances. Yet, because it disregards later-life social accomplishments and status, this steadiness may be disadvantageous [28].

In the urban and rural context, SC and ST women in rural area have a higher probability of being underweight, whereas in urban area non-underweight are in high prevalence, showing a consistency with previous researches. In light of these findings, policies addressing non-underweight and chronic disease in LMICs should not only measure mean levels of risk factors at a national level, but should also assess the implications of on-going changes in diet and physical activity on health disparities, including disparities within urban areas [29].

Similarly, several studies showed that as women aged, their rates of overweight and obesity considerably increased [30]. In West Bengal, SC and ST women showed an increase in non-underweight as they aged, according to our study.

The present study has also identified the role of food and dietary factors in determining the nutritional status of SC and ST women, which is consistent with the findings of previous research [29, 31–33]. Furthermore, the study has highlighted the need for targeted interventions that address socio-economic disparities and promote a healthy and balanced diet to reduce the gap and improve the population's nutritional status, which is consistent with the policy implications of earlier studies [27, 34–38].

When measuring nutritional status, it might be difficult to classify people because the kind of food diversity that a household consumes everyday more or less depends on their nutritional status. Unless we have comprehensive evidence to support this argument, religion should at the very least play a supporting role. Yet, diverse ritualised food preparation procedures will likewise have little effect on nutrition. Economic, health, medical, food, and other facilities were more readily available in urban regions than in rural ones. As a result, individuals in most urban areas are eating more food due to greater economic strength and buying power parity than in rural areas, which has an impact on nutrition. One important aspect of nutrition is wealth. Due to belonging to the poorest category, those individuals are less economically solvent, which has an immediate impact on their daily food consumption because low-income results in low purchasing power, which leads to an increase in the number of overweight individuals [24].

Regarding the methodology, the present study employed multivariate decomposition analysis to identify the factors contributing to the nutritional gap between SC and ST women. This method allowed for the examination of the relative contributions of various factors to the nutritional gap and provided essential insights into the factors that policymakers should prioritize when developing interventions to improve the nutritional status of SC and ST women. Overall, decomposition analysis can provide valuable insights into the complex factors that underlie nutritional disparities among SC and ST women in West Bengal and can help inform policy decisions aimed at addressing these disparities.

The present study has contributed to the existing literature by providing a comprehensive analysis of the factors that determine the nutritional status of SC and ST women in West Bengal. The findings suggest that addressing socio-economic disparities and promoting a healthy and balanced diet could reduce the nutritional gap between SC and ST women. The study also emphasizes the importance of promoting educational equity for historically marginalized groups such as SCs and STs. The policy implications of the study highlight the need for targeted interventions that address the significant factors identified in this study, such as wealth index, education, place of residence, and food and dietary factors. Therefore, interventions aimed at improving the highlighted characteristics of SC and ST women could help address the issue of undernutrition and improve their health and well-being. Such interventions could include targeted policies and programs that focus on improving the economic and educational opportunities available to these women.

## Conclusion

In conclusion, the research paper highlights that the study shows a significant population suffering from underweight in West Bengal. The spatial analysis reveals that the prevalence of underweight women is common throughout the districts, with Purba Barddhaman (8.95%) and Paschim Medinipur (8.84%) having the highest prevalence of underweight reproductive SC women in the total SC population, and Purulia (16.41%) and Jalpaiguri (15.93%) having the highest prevalence of underweight reproductive ST women in the total ST population in West Bengal.

The multivariate decomposition analysis presented in the research paper identifies the factors contributing to the nutritional gap between SC and ST reproductive-aged women in West Bengal. The study suggests that socio-economic factors, particularly wealth index, education, place of residence, and age group, significantly contribute to the gap. Food and dietary factors, such as the consumption of fish, fruits, aerated drinks, and fried foods, also significantly contribute to the gap.

The policy implications of the research paper suggest that to reduce the nutritional gap between SC and ST women, and policymakers should address socio-economic disparities by improving access to education and employment opportunities, particularly in rural areas. Promoting a healthy and balanced diet is essential to increasing the availability and affordability of nutritious foods, mainly fish and fruits while discouraging the consumption of aerated drinks and fried foods.

In addition, policymakers can promote the consumption of pulses, beans, and eggs by providing subsidies or incentives to farmers, promoting the cultivation of these crops, and increasing awareness among the population about their nutritional benefits.

Overall, the research paper's findings provide essential insights into the factors contributing to the nutritional gap between SC and ST reproductive-aged women population in West Bengal, and the policy implications highlight the need for targeted interventions that address socio-economic disparities and promote a healthy and balanced diet to reduce the gap and improve the population's nutritional status. However, it is important to note that the study's limitations include its focus on undernutrition status and socio-demographics, dietary and food habits, smoking and alcohol consumption behaviour and geographic region taken as independent variables only. No comparison analysis was carried out across all population groups to understand the vulnerability risks of these domains. Future studies could consider exploring these domains in greater depth to provide a more comprehensive understanding of the factors contributing to the vulnerability of SC and ST populations.

## Data Availability

The study is based on secondary data analysis. No data was collected for this study. The datasets generated and/or analysed during the current study are available in the DHS website (NFHS-5) (The DHS Program—India: Standard DHS, 2019–21 Dataset).

## Abbreviations

SC: Scheduled Caste
ST: Scheduled Tribe
BMI: Body Mass Index
SES: Socioeconomic status
LMIC: Low- or Middle-Income Country
